# Antenatal preparation as care: birth stories and collective learning at work

**DOI:** 10.3389/fgwh.2025.1592538

**Published:** 2025-06-30

**Authors:** Leah De Quattro

**Affiliations:** Centre for the History of Science, Technology and Medicine (CHSTM), The University of Manchester, Manchester, United Kingdom

**Keywords:** birth stories, antenatal preparation, childbirth education, care, collective knowledge, socio-narratology, group-led, feminist science and technology studies

## Abstract

Distressing and harmful birth experiences are the norm even in well-resourced countries, and conventional antenatal education struggles to adequately prepare birthing people. Drawing on previous research in support of participant-led antenatal education, a recent UK-based ethnographic study asks how birthing people use collective practices to produce birth knowledge. Data comes from participant observation at 24 antenatal sessions (*n* = 201) including conventional classes and community-based groups, plus 5 interviews with session leaders. The researcher analysed data using a novel application of template analysis, framed by feminist technoscience, ethnography and socio-narratology. Findings show how group-led sessions, storytelling and other collective knowledge practices *take care* of birthing people. Several facets of care emerge from this inquiry, such as materiality, emotionality, working athwart dominant narratives and creating “care-full” absences or spaces. Excerpts from antenatal preparation sessions specifically demonstrate various approaches to knowledge working – and caring – in practice. A focus on real-life examples and implications ensures findings are useful and relevant for birthing women, midwives, antenatal educators, institutions, policymakers and more.

## Introduction

Distressing and harmful birth experiences are the norm even in well-resourced countries like the UK (1, 2), and poor outcomes are significantly more likely for black women and other people of colour (3, 4). Meanwhile, conventional antenatal education struggles to adequately prepare birthing people, as seen in surveys of mothers who felt uninformed or unprepared for birth (5, 6), especially minoritised communities and younger mothers (7). In this paper, I present findings from *Knowing Childbirth*, a UK-based ethnographic study that explores collective learning as a potential solution to worsening birth experiences (6) and wider maternity care crises (1, 2, 8).

### Contemporary antenatal education

Childbirth preparation is commonplace is modern Britain. However, a recent Care Quality Commission survey suggests only one-third of pregnant mothers attended classes (9), and most studies were inconclusive as to benefits (10–15). Still, many women speak positively about antenatal classes (9, 15–18), with two key benefits—support (5, 15, 16, 19) and information (5, 20, 21)—repeatedly featuring in previous research.

Inconsistencies in existing literature prompt questions about shortcomings in standard childbirth education. In part, busy lives and funding cuts may mean shorter classes, with less time available for building communities or engaging in complex learning (16, 21, 22). Information was also a fraught concept, as classes either reinforced or challenged biomedical norms (10, 22, 23), tended to promote institutional compliance (10, 21, 23), offered too much or too little information (12, 18, 21), or presented content that did not match lived realities (24, 25). Lower uptake and satisfaction with antenatal classes among black women and women of colour (26) may relate to transport, cost, language, time and other practical obstacles (18, 26, 27), as well as culturally inappropriate information (15, 26), negative stereotyping (18, 27), and hegemonic norms promoted in classes (20, 22, 26–28).

### Collective learning and birth storytelling

Despite some studies' tentative findings, the National Institute for Clinical Excellence clearly recommends “participant-led” antenatal preparation (12). Several studies suggest that collective approaches (e.g., group-led discussion, storytelling) help birthing people by broadening expectations and building relationships (10, 29–32), although this research is scarce and at times ambiguous (33, 34). However, *Knowing Childbirth* found little evidence of this format in standard NHS or NCT classes.

Birth storytelling is a key component of collective antenatal learning, and appears in many studies about antenatal preparation [e.g., (5, 10, 16, 20)]. Other studies engage with birth stories to learn about childbirth generally [e.g., (11, 19, 20, 23)]. Another significant area of research is the impact of birth storytelling on the teller [e.g., (35–37)], which includes mixed reviews on the efficacy of postnatal “debriefing” alongside calls for better postnatal listening services [e.g., (38, 39)]. Only very few studies [(31, 34, 35)] specifically investigated the educational impacts of birth stories, and this research addresses that gap.

Previous research emphasises that effective birth knowledge requires people to navigate conflicts and complexities (13, 16, 20) without undermining individuals or relationships (40, 41). *Knowing Childbirth* engaged understudied birthing subjects and antenatal settings to explore how storytelling, group-led discussion and other collective knowledge practices help to meet this call. This study defined collective learning as drawn from lay knowledge, personal histories or group discussion. These practices contrast more formal or top-down practices, such as evidence-based “authoritative knowledge” or guideline-based “procedural knowledge”. While all knowledge is collective to some extent due to the role of listener interpretation (42, 43), this research distinguishes between knowledge that inclines toward collectivity and practices that reinforce hierarchy.

### Care, birth and knowledge

Notions of care permeated the study due to links among birth, mothering (44), midwifery and other birth-related caregiving (45). Care—like mothering and midwifery—is vital, undervalued, joyful, mundane, radical, oppressive, physical and deeply affective (44). *The Care Manifesto* defines care as physical, emotional and social practices that nurture “the welfare and flourishing of life” in part by embracing interdependence (46). Birth knowledge is one of these practices.

Much academic and activist literature on care relates to birth. Decades of feminist science and technology studies scholarship reinforce the materiality, emotionality, relationality and multiplicity of care (47–49). Good care is “persistent tinkering” (50)—with the word “tinkering” emphasising a responsive and material practice, rather than a homogeneous or ethical ideal. For a labouring woman, tinkering could include offering food, a hot bath, an epidural or supportive silence; in an antenatal group, it may be discussing what to bring to hospital or how to hire a birth pool. Other scholars elaborate the affective-effective nature of care, as simultaneously a physical practice *and* an emotional, symbolic labour (47, 49). The results and discussion section demonstrates positive examples of encouragement from caregivers during labour and pregnancy—as well as the reverse, when inadequate emotional support manifested poor care.

Another well-recognised aspect of care in birth and beyond—even when carers mean well—is a “dark side” that reinforces norms or demands compliance (46, 51). For example, birth knowledge often evokes essentialising and patriarchal natural birth narratives (52) or “guideline-centred” rather than person-centred medical care (41). Scholars also note the variability of care due to differences in ethnicity, geography or other factors (51). Discrepancies in birth care and knowledge emerge in differential rates of childbirth injury and death among racialised women and children (3), or the underrepresentation of people of colour in British antenatal education (7). To enact good care, caregivers must recognise the potential downsides and inequities of some care.

Understanding the multiplicity of care enables a rich analysis of collective birth knowledge practices. Following a brief introduction to study design and data, this paper explores how birthing people engaged with collective knowledge. Drawing on materiality, emotionality and other facets of care that emerged during analysis, the results and discussion section interrogates the work of specific knowledge practices. Findings establish a strong foundation for the idea that collective birth knowledge *takes care of* birthgivers, birth workers and beyond.

## Methodology

Theory and methods, alongside a novel, manifold approach to thematic analysis, mirror the subject of research with a commitment to multiplicity, context and co-construction. *Knowing Childbirth* occupies a posthumanist feminist ethnographic stance, embedded in its geo-socio-temporal position, as well as a partisan focus on reducing inequities and improving lives. This situated, holistic approach enables credible and useful findings for birthing people and those who care for them.

Data collection took place in two stages, a pilot study (2016) and the main study (2019–2020), which formed the basis of ESRC-funded MSc and PhD (53) dissertations, and related articles (32, 54). All research received ethical approval from the university or the NHS, and all participants gave signed, informed consent.

### Theoretical framework

In form, as in content, this project utilised posthumanist feminist and critical theories that challenge assumptions about “objective” knowledge and individual subjectivity, as found in feminist science and technology studies. Feminist posthumanism acknowledges that physiological, emotional, social, economic, geographical, technological and other contexts co-constitute reality (47–49, 55). This multiplicity renders all knowledge as partial and situated, and human subjects as complex, dynamic and relational—albeit still grounded in specific bodies (47–49, 55). Similarly, feminist ethnography positions qualitative research as an active process where researcher and participants co-construct meaning, all perspectives are partial—including insiders, researchers and participants—and truth is fluid, incomplete and polyphonic (56). This contingency and multiplicity is not weakness, but a source of richness, depth and meaning (55, 56). Socio-narratology builds on this approach, again emphasising polyphony in the form of multiple, contradictory meanings and overlapping contexts (43). As a mother learning about the experiences of childbearing women, I explored ethnographic conundrums around body knowledge (57), feminist solidarity (56, 58) and insider research (56, 58). Awareness of researcher reflexivity corresponds with the wider theoretical approach, centred on interdependencies among knowledges, participants and wider contexts.

### Recruitment and data collection

The theoretical framework justified participant observation at antenatal sessions as the primary method of data collection, as a context-rich, polyphonic and grounded approach in keeping with collective learning (56, 58). Other data sources included a handful of semi-structured interviews with session leaders, and a participant questionnaire to collect demographic information. Note, the importance of demographic detail emerged during pilot study analysis, and thus only the main study included the questionnaire. In addition, I kept a log to monitor my own partial perspectives, including initial ideas from antenatal sessions and interviews, reflections just after data collection and thoughts that arose during analysis.

*Knowing Childbirth* relied on purposive sampling, as participants joined the study by virtue of the fact they attended or delivered the relevant antenatal preparation sessions. I recruited participants by first seeking approval from gatekeepers to attend antenatal preparation sessions, or to conduct interviews with midwives and educators. All participants received study information and consent materials in advance and in person, with chances to ask questions and decline participation. Eligible participants were at least 18 years of age, proficient in English, and attended an observed antenatal session, including pregnant women, postnatal mothers, partners, midwives, teachers and facilitators. Interviewees were professionals or volunteers who delivered observed sessions.

At each antenatal session and interview, I collected as much verbal and non-verbal data as possible by audio-recording where permitted, in addition to hand-written notes for nonverbal observations (e.g., setting details, props, gesture, tone). During antenatal sessions, I only spoke when engaged by other participants, namely two sessions when I was visibly pregnant during the pilot study, and two NHS Homebirth classes where the midwife-teacher invited me to tell a birth story and answer attendee questions. For the interviews, I used a broad topic guide to gather organisational information and other practical details, as well as professional perspectives on antenatal sessions. Additional questions developed from ongoing participant observations, secondary research and interviewee input. Recording and data handling took place in accordance with ethical guidelines and university policies, including pseudonymisation of all participants and removing identifying characteristics.

### Coding and data analysis

To analyse 50 h and nearly 300,000 words of transcripts, I first organised the data according to template analysis, a form of thematic analysis (59). Beginning with themes related to the research questions, I read and re-read the transcripts, coding and re-coding, adding, discarding, consolidating and rearranging themes and subthemes to build a clear, concise and comprehensive template (59). I coded transcripts comprehensively, in order to clearly see and compare macro-level prevalence and interactions among themes using a technique developed during the study. Utilising NVIVO matrix queries, I generated tables depicting the prevalence of themes by word count, usually by format (teacher-led or group-led). After converting these tables to percentages (of the total word count) in Microsoft Excel, “heat map” formatting emphasised higher and lower numbers. By the same process, I constructed tables to visualise overlaps among themes, and compared to overall prevalence of a given theme, or in a different setting. Notably, these thematic tables were not research findings, but rather tools to focus the gaze for subsequent qualitative analysis. By illuminating some of the clearest correlations and absences, this initial stage indicated trends worthy of further investigation, decreased the influence of researcher interpretation and made findings more meaningful.

Next, I sought to complicate and locate trends in the thematic tables within specific excerpts, geo-socio-temporal contexts and existing literature. Some techniques from conversation analysis proved useful, including attention to small verbal and nonverbal details, and explicit or implied assumptions (60). However, analysis more often took a content-focused, relational stance that prioritised insider knowledge, while continually considering contexts (e.g., audience, settings, socio-economic structures), silences and polyphony (e.g., multiple voices and meanings), as in feminist technoscience (55), feminist ethnography (56) and socio-narratology (43). This manifold analytic approach continually switched perspectives—“zooming in and out” from the data—developing critical and robust findings.

## Results and discussion

Antenatal session transcripts demonstrated how attendees engaged with storytelling, group-led sessions, other collective practices and birth knowledges in general. Interviews with midwife-teachers and group facilitators added contextual information and practice-based perspectives. Collective learning appeared in all antenatal preparation settings as storytelling, intuition, comparing, questioning, humour, group-led interpretations of formal knowledge, and more. To contextualise the discussion below regarding how people used collective learning to learn about childbirth, this section first summarises research settings, participants and the thematic template.

### Research settings

Across the entire study, I carried out participant observation at 24 antenatal sessions, plus 5 interviews with midwife-teachers and other facilitators. Observed sessions included 6 National Health Service (NHS) standard classes, 3 NHS homebirth classes, 6 National Childbirth Trust (NCT) classes, 5 community-based Positive Birth Movement (PBM) groups and 4 community-based homebirth groups.

Antenatal settings differed significantly in format, time of day, setting, duration, number of participants and themes. Research took place in a number of locations in cities in the north of England, according to existing arrangements or interviewee convenience, and including maternity hospitals, SureStart centres, libraries, community centres, cafés, churches and homes. Antenatal preparation sessions lasted from one to three hours and included between 4 and 33 participants, excluding the researcher.

The NHS delivers the most commonly attended antenatal classes in the UK (19). Standard NHS classes tended to cover similar curricula (i.e., physiological birth, pain relief, labour “complications” and life with baby). NHS midwife-teachers used standardised teaching aids, a fairly consistent curriculum and a hospital-specific film about pain relief options. NHS Homebirth classes formed part of an effort to increase homebirth rates, taught by willing community midwives alongside their normal workload. These sessions followed a looser format, including an overview of the *Birthplace Cohort Study* (2020), a description of procedures regarding homebirth, a homebirth story where possible, and answering questions from attendees.

Previous studies have suggested that the NCT offers the most second most-popular antenatal education option, at a cost (20). In observations, NCT course materials and curricula appeared standardised, with similar topics to standard NHS classes. However, NCT classes typically involve more sessions, postnatal information, and partner involvement (5). During research, teacher Frances developed an air of informality in her classes, which included telling personal stories and encouraging attendee input. While the NCT originally promoted nonmedicalised birth, today's organisation relies on promotion of parental choice and scientific evidence to build brand identity, educational programmes and campaign messages (61).

Group-led Homebirth and PBM sessions varied significantly in format and content depending on attendee input. However, almost all sessions shared a focus on attendee birth stories and questions, and most included some discussion of homebirth and reducing medical interventions. Regular groups usually developed as individual initiatives, with PBM groups affiliated with the wider PBM. The PBM works to empower birthing people and resist patriarchal medical practices and obstetric violence, also sometimes reinforcing hegemonic ideas of happy, gender-normative, white, middle-class birth [e.g., PBM Welcome Pack imagery (62)].

### Participants

The entire study engaged a total of 201 participants, including the researcher, 43 participants from the pilot study and 157 participants from the main study (Table 1). Around two-thirds joined the study via NHS or NCT classes, and one-third contributed as part of a community-based group. Participants were two-thirds female—of which two-thirds were pregnant—and one-third male. Two-thirds of participants were first-time parents and one-third already had children.

**Table 1:** Summary of all participant attributes (*n* = 201).

Attribute	Proportion of responses
Type of session	34% group-led, 66% teacher-led
Sex	66% female, 34% male
Previous children	35% yes, 63% no
Pregnant	66% yes, 33% no (of females)

As noted above, only participants in the main study gave other demographic details (Table 2). Just over half of main study participants were 25–34 years of age, one-quarter were 35–44, and the remainder under 25 or over 45. Almost all participants were married or cohabiting, spoke English as a main language, did not have a disability and had a current occupation. Regarding race/ethnicity, 76% identified as white, 9% as Asian/Asian-British, 7% as mixed and 6% as black/black British. As for education, participants split roughly into one-third with postgraduate degrees, one-third with university Bachelor degrees and one-third with A-level, GCSE, Diploma or Entry-level qualifications.

**Table 2:** Details of main study participant attributes (*n* = 158), excluding “no answer”.

Attribute	Percentage of responses			
Session type	72% teacher-led	49% NHS standard	23% NHS homebirth	28% group-led
Sex	67% female	33% male		
Previous children	66% no	31% yes		
Pregnant	31% no	68% yes	(% of females)	
Age	7% under 25	60% 25–34	26% 35–44	7% over 45
Partnership	46% married	44% cohabiting	9% single	
Ethnicity	76% white	9% Asian/British	7% mixed	6% black/British
Main language	91% English	8% other		
Disability	97% no	1% yes		
Occupation	88% employed	6% student	3% unemployed	
Qualification	35% Bachelors	32% Postgrad	16% A-level, Dip	9% GCSE, Entry

### Thematic template

During the coding stage of template analysis, several groups of themes arose from the transcripts: topics (content or “what” people talked about), types and techniques of knowledge (form or “how” people presented information), and integrative themes that acted multiply as topics and knowledge practices (Table 3). Examples of topics included stages of labour, labour techniques, other people (e.g., partners, midwives) and experiences of selfhood (e.g., emotions, pain). Knowledge types roughly divided into formal (e.g., evidence-based “authoritative knowledge”, guideline-based “procedural knowledge”) and informal knowledge (e.g., different types of stories, intuition). Many techniques for navigating knowledge also appeared, such as humour, “comparing” or emphasising difference, “normalising” or emphasising similarity and silence. Variations on control (e.g., choice, compromise, uncertainty, chaos) and dis/trust served as crucial integrative themes, running throughout the data.

**Table 3:** Thematic template with main themes and abridged subthemes.

Topics of discussion	Knowledge types	Knowledge techniques	Integrative themes
Labour stages	Formal – Authoritative – Procedural – Quantitative	Questioning	Variable trust – Trust – Distrust
Logistics		Explaining	
Risk		Demonstrating	
Other actors (e.g., baby, midwife, partner)		Comparing	
		Normalising	
Self-experience (e.g., body, pain, emotion)	Informal – Intuition – Stories: First-hand, second-hand, distant (e.g., media, third-hand)	Humour	Variable control – Control (e.g., agency, choice, resistance) – Compromise (e.g., interpersonal, change) – Chaos (positive or negative)
Labour techniques – Non-medical (e.g., active, relaxation, support) – Medical normal (e.g., Monitoring, Entonox) – Medical complex (e.g., pharmaceutical, Caesarean)		Silencing	

### A summary of knowledge practices in antenatal settings

This discussion next summarises how knowledge practices appeared in the data, and how collective approaches demonstrated care. Subsequent sections elaborate how particular collective knowledges performed care in practice, including less common collective approaches (i.e., stories in classes, second-hand and more distant stories, intuition), and more pervasive techniques (i.e., comparing, normalising, humour, silencing). Finally, a single story demonstrates how in-depth storytelling utilised a range of collective knowledges to care for birthing people and more. A brief summary of how knowledge practices appeared in the data contextualises the results and discussion (Table 4). Group transcripts primarily comprised informal knowledges, with lots of first-hand stories, some second-hand stories and occasional distant stories or intuition. Group participants also used a small but significant proportion of less collective, formal knowledges, including authoritative, procedural and quantitative knowledges. Main techniques for navigating knowledges in groups included comparing, humour and explaining; normalising, questioning, demonstrating and silencing were less typical.

**Table 4:** Summary of differences between groups and classes.

	Similar in groups and classes	More in groups/less in classes	Less in groups/more in classes
Knowledge types	Intuition rare	Informal, especially story (esp. first-hand)	Formal, esp. procedural
Knowledge techniques	Questioning and silencing rare	Comparing, humour	Normalising, explaining, demonstrating
Integrative themes	Chaos rare, usually negative	Control (incl. resistance), compromise and chaos Trust and distrust	Choice

Teacher-led sessions showed the reverse trend: Formal knowledges dominated classes (mainly procedural, often authoritative and sometimes quantitative) along with less collective techniques for navigating knowledge (i.e., demonstrating, normalising and explaining). However, informal knowledges and more collective knowledge techniques (e.g., story, intuition, humour, comparing, silencing) also featured in classes. Regarding control and trust, classes usually reinforced control, choice and trust, while group-led sessions presented a much wider variation on these themes.

### Collective knowledge as care

Well-recognised attributes of care repeatedly arose with relation to collective knowledge, as group-led discussion and storytelling attended to physical *and* emotional realities, and sometimes worked to remedy “dark sides” of conventional birth knowledge. Stories, intuition and comparing “tinkered” by adding experiential nuance and materially grounded detail to formal knowledges. Emotional resonances, usually via stories or humour, amplified non-normative experiences and ambivalence. In-depth storytelling proved a particularly care-full form of knowledge. Stories, especially when told in group-led settings and enriched by listener and teller interaction, included a wide range of collective practices—humour, comparing, normalising, silences, and bits of intuition as well as authoritative knowledge.

Other aspects of care—less well-represented in previous literature—arose in creative responses to conflict, complexity or distress. Many birth stories include moments of care where midwives and mothers trod a path between compliance and noncompliance [e.g., (41)], in order to “work athwart” (63)—meaning sideways, rather than contrary or parallel—standard procedure. Collective knowledge practices also worked athwart people, institutions, expectations or other knowledges, presenting care-full alternatives to acquiescence or opposition. As shown below, intuition could accommodate the unexpected, humour disrupted taboos, and stories and group-led discussion situated formal knowledges in the birthing person's subjective experience.

Transcripts also revealed the existence of important, care-full gaps in maternity care and birth knowledge. Care literature focuses on attentiveness [e.g., “tinkering” (50)], but many participants preferred a hands-off approach from midwives or avoided hearing about certain birth outcomes. In observations during research and personal life as a parent, good care often included stepping back, giving space and a perceived—or actual—inattention. The caring gaze, or too much information, might oppress, limit or undermine (47, 48). I explore below how absences in care and knowledge could be productive, as space afforded chances to perform self-care, consolidate knowledge, build trust, deprioritise control or level caring relationships. Silences in birth knowledge could also reinforce taboos or compliance, or express uncertainty, and some birthing people chose disengagement—intentional silencing—as a protective technique.

Useful antenatal preparation must navigate conflicts and complexities (13) without undermining individuals or relationships (41). *Knowing Childbirth* found that collective learning meets this complexity with care and connection, tinkering with and grounding abstract knowledges, attending to emotional resonances, building alternatives by working athwart norms, and even producing care-full spaces around certain topics.

### Uncommon collective practices: complementing standard knowledges

Informal group-led settings engaged more collective learning overall, and formal teacher-led classes utilised fewer collective practices. This section investigates more exceptional collective practices, namely stories in classes, non-first-hand stories and intuition.

#### Class stories: materiality, affect and disrupting expectations

Some stories complicated the binarised picture of storied groups vs. formal knowledge-based classes. Storytelling did occur in classes, with main topical associations including transition, antenatal preparation, relaxation techniques or partners. These overlaps seemed to reflect lay accessibility, less biomedical relevance, and also perhaps that certain topics not only allowed for storytelling, but called for it. In a storied description of transition, midwife-teacher Justine tinkered with broad-brush understandings by explaining in detail what a birthing woman might experience, and why:

¨Justine: When you suddenly get contractions, and pressure in your back passage, you don't like it. Okay? And for about 5 min, a lot of women, lose it. Just because, they, they are not used to the sensation of both together … And if you're a birth partner within punching distance you'll get hit. <Laughter>” … It's very scary for the woman, but after a couple of minutes, it's over … And then you suddenly go, <clicks fingers> push! … You're so focused on pushing, it takes over your whole body and you forget about all the pain.

Her holistic, affective-effective account acknowledged both physical and emotional concerns, using humour to evoke laughter that mediated the hypothetical woman's distress.

Another example appeared in a crowded NHS class with over two dozen attendees, as a pregnant woman and the midwife-teacher exchanged anecdotes about pain relief:

Cassie: So that'd be the one where they say, they can't really remember? Because they're drowsy? (Olivia: Ahh –) Well just, after it, I've just heard people saying that, they had, pethidine, that it's all a bit fuzzy, that, during the, baby's born, and everything after it's all a bit fuzzy with –

¨Olivia: All a bit fuzzy? Ah, maybe. I'd say more so with pethidine than with diamorphine and that's the reason it changed, actually, so. Eh, generally we find that we prefer diamorphine, pethidine made everybody a bit woozy. But I had it with my, my first is a bit older and em, I enjoyed pethidine, I have to say <Laughs> I really like it! But, you know, everybody's different.

This excerpt features different stories, facilitated by knowledge techniques of comparing and humour, which grounded generalised knowledge about diamorphine. Cassie's second-hand story converted an earlier comment that diamorphine could make you feel “a bit out of it” into amore embodied reality: New mothers might not remember much about the first moments with their baby. Midwife-teacher Olivia confirmed that for that reason, standard practice had changed from offering one opioid (pethidine) to another (diamorphine). However, she did not limit her reply to a procedural point, but introduced her own story that emphasised a very different possibility [“enjoyed”, “really like(d)”] regarding opioid analgesia and childbirth. As she performed her recollection of pleasure for the class, with laughter adding an affective lightness, Olivia's story challenged predominantly negative expectations about birth and heavy pain medication. Her humour might have buoyed attendees in an otherwise fairly difficult bit of the class that included serious faces, loaded silences, muttering between couples and only a few spoken concerns. At the same time, as explored later, this moment of humour silenced or diverted residual concerns about pharmacological intervention.

Participant stories could present more care-full knowledges regarding difficult or complex aspects of childbirth, as hinted at in previous research (29, 30, 31, 36). Storytelling is a flexible, entangled mode of communication that inherently tinkers—adds grounded nuance—as tellers, audience and settings co-construct accounts (42, 43). By incorporating a range of narrative devices and other knowledge techniques as needed, stories offered holistic and multiple approaches to information, although humour bypassed as well as mitigated concerns. In its attention to material and affective gaps in formal knowledge practices, class-based storytelling could disrupt dominant discourses, diversify expectations and increase engagement.

#### Further removed stories and distancing concerns

Like stories in classes, second-hand and more distant stories in all settings helped to address problematic topics. These stories associated more than normal with complex medical interventions, negative emotions and chaos. This distance constituted a care-full, protective space while engaging with negative expectations. Second-hand and more distant stories enabled participants to acknowledge difficult aspects of birth without uncomfortably close engagement. The affective impact of second-hand and distant stories was central: Claire drew reassurance from a second-hand story of induction, Jade felt distress at stories of unwanted Caesarean births, and Naomi responded negatively to a hypothetical medical emergency. Where formal knowledges or first-hand stories might be scarce, insufficient or uncomfortable, further removed stories provided additional resources to manage participants' affective concerns—not ignoring nor dwelling on difficult topics, but taking an oblique approach.

One example arose at a PBM session from Noelle, a midwife and attendee at the group. She recalled a woman she supported whose chaotic-sounding birth included a scheduled and cancelled Caesarean, an attempted and failed induction, and an eventual Caesarean birth. In part, this tale enabled a double vision of hospital induction, situating abstract procedure in one woman's specific reality. Perhaps more importantly, Noelle's account worked athwart negative expectations (the painfulness of induction, or distress amid escalating hospital interventions) by underlining how the woman participated in her care decisions, and the positive affective result (“She was, delighted”). Sharing this story offered listeners a similar emotional buoyancy regarding less desirable birth experiences, with practical suggestions around the importance of good communication and compromise. By enabling people to engage with difficult topics—but not so closely as to cause discomfort—further removed stories played an important function in antenatal preparation sessions. Often performing a negative affective role observed in other studies (33, 34), more distant stories also overcame taboos and broadened expectations.

#### Intuiting labour: embodied, oblique knowledges

Intuition bears close consideration as a collective, informal knowledge practice that only appeared rarely in the antenatal sessions. This lay-accessible, subjective, embodied knowledge type interacted with multiple knowledge practices in group-led settings, often disrupting formal knowledges. Intuition in groups linked thematically to dis/trust and variations on control, while classes tended to associate intuition more narrowly to trust and letting go of control. Intuition in classes also seemed topically limited to pushing, active birth techniques and bodies. Groups much more broadly engaged intuition regarding different stages of labour (antenatal preparation, early labour, established labour, pushing), non-medical (relaxation, active birth, interpersonal support) and medical (monitoring, diamorphine) labour techniques, risk, other actors (baby, midwife, hospital/doctor) and self-experiences (mostly body but also negative and positive emotion).

References to intuition strongly evoked care in all settings. Even within the narrow remit afforded to intuition in classes—when midwife-teachers encouraged pregnant attendees to listen to their own bodies, let go, and push their babies out how and when it felt right—these comments care-fully departed from conventional narratives. Justine offered this guidance about pushing to her NHS antenatal class:

Justine: So, em, a lot of women feel they have to be told? When to push, in labour? But that's not true. What we encourage you to do, is just breathe and breathe and breathe, until the point where you physically can't stop yourself from pushing, and your body will just take over, and it'll make you push. You don't need to push to deliver your baby, your body will do it for you.

Justine's description both welcomed and carefully boundaried the role of intuition. She began by revaluing innate knowledge, stating unequivocally that it is “not true” that women “have to be told” to push. After a brief caveat where she asked listeners to ignore their intuitive desire to push by breathing instead, like midwives in other research (40), she reaffirmed bodily intuition as trustworthy and correct. As in other excerpts that attempted to verbalise the physical intensity of childbirth, Justine conflated the birthing person and her body, and switched between third-person “your body will do it for you” and more active “you’ll just, push”. However she presented birthing subjectivity, intuition emerged as clearly—yet contingently—important.

The use of intuition regarding pushing seemed to respond to a gap in formal knowledges, which did not satisfactorily attend to the powerful corporeality experienced by birthing women in this research and other studies [e.g., (28, 29, 31)]. Justine curated space for this knowledge practice, describing in detail how people might experience or trust their intuitive sense to push. Intuition worked athwart dominant assumptions in institutional settings—that professionals provide the most credible childbirth knowledges (10, 22, 23)—not by undermining formal discourses but offering an addition. Within boundaries that reinforced institutional knowledge even as she challenged it, Justine care-fully presented intuition as a trustworthy source.

While some teacher-midwives encouraged partial confidence in intuition, many group excerpts placed intuition at odds with medical caregivers. Kath told the following story at a PBM group that she facilitated:

¨Kath: [My friend] said to me, one thing I'll say to you now, “Don't let them tell you not to push.” She said, “I'm sure the reason I had a good back-to-back birth was because I didn't have some dickhead midwife telling me not to push.” <Laughs> <Sounds of assent> And as soon as my midwife walked in for my birth, and it was a midwife I'd met prior, I went, don't tell me not to push! Because I'm going to push! And I've been told I'm not allowed to - you know, straight off, I'm gonna push, I'm gonna push. Because you can't not if that urge is there.¨

Kath's story corresponded and deviated from Justine's approach to intuition and pushing, although both women's comments drew on their expertise as trained midwives *and* birthgivers. Like Justine, Kath offered grounded information about the importance of intuition in facilitating birth. However, Kath rejected professional boundaries on intuition and prioritised subjective knowledge in communication with caregivers. Her privileged status as a midwife enhanced her critique of standard practice in this and other excerpts; she wielded lay and professional authority. Within her story, the affective power of humour and colourful language took the sting out of her obstinacy and made her resistance to professional instruction more permissible. Similarly, many other group examples of intuition added real-life nuance, variability and contingency to people's behaviour during childbirth and disrupting medical knowledges.

Intuition appeared at both ends of the spectrum of control in groups—from reclaiming control by resisting authority, to letting go of control—and both associations worked athwart hegemonic notions of control and knowledge. As a form of resistance, excerpts about intuition often presented the embodied self as an alternative source of control and knowledge. For example, Tanya, Sue, Mandy and Esther valued innate knowledge of labour over quantitative, procedural measures of cervical dilation. Nikita's pleasure at vocalising intuitively during labour disputed prevailing ideas about childbirth as suffering, and of docile patienthood and femininity: “I like let out this orgasmic sound … really in contrast with the like, deep roaring … But it is good just to be able to let go, and like, go with it”. Where intuition linked to letting go of control, it upset norms of subjective control. Several homebirth stories emphasised the contingency of knowledge and agency during birth: Sana plaintively recalled “I didn’t know, what to do … where is my inner wisdom?”; Rosa claimed, “I didn’t feel like I pushed at all. He just was born”. Intuition contested dominant notions of subjectivity, decentring the birthing subject and/or engaging “the body” as the primary active agent.

Altogether, intuition offered a useful, care-full collective resource in antenatal preparation sessions, and other research supports the importance of this knowledge practice (28, 31, 40). Intuition adjusted authoritative or procedural perspectives by attending to the lived reality of childbirth. Especially in group settings where intuition implicated a wider range of topics, this knowledge practice offered additional information to formal knowledges, mainstream birth narratives, behavioural norms and notions of subjectivity. Intuition often associated with techniques such as humour or normalising in its knowledge-as-care work, explored further in the next section.

Less common collective knowledge practices—unexpected stories, intuition and lay use of formal knowledges—performed important care work in antenatal preparation sessions. Excerpts demonstrate how class stories added materiality to diversify and ground abstract formal knowledges, as well as emotionality to encourage engagement. More distant stories engaged difficult aspects of childbirth by acknowledging without approaching too closely, keeping concerns at a distance. Intuition operated an embodied practice that provided nuance and alternatives to normative knowledges and other sociocultural narratives. Alongside more typical collective knowledges explored in the next section, these unusual collective practices cared for birthing people and knowledge.

### Navigating with care: comparing, normalising, humour and silencing

Techniques for navigating knowledge care-fully permeated the antenatal preparation sessions, mediating, elaborating and responding to various claims. While groups mainly used more collective techniques (e.g., comparing, humour) and classes tended towards less collective approaches (e.g., explaining, demonstrating), all these knowledge techniques appeared in all types of sessions. This section focuses the most common collective knowledges: comparing, normalising in group settings, humour and silencing. These practices not only amended other birth knowledges, but affectively entangled birthing people, disrupted prevailing or conflicting discourses, and created care-full epistemic spaces. I also considers dark sides to this knowledge-as-care, as some techniques could bolster dominant ideologies, encourage compliance or suppress deviation from sociocultural norms.

#### Comparing: tinkering, working athwart and individualising

Comparing emerged as the most utilised knowledge technique in groups, foregrounding contrasting maternity experiences, opinions, claims or practices that appear throughout existing literature (17, 18, 22, 26, 29). Although teacher-led settings tended toward normalising (i.e., reinforcing similarity), comparing also arose frequently in classes. Through the lens of care, comparing signified a crucial technique for adding materiality and nuance to birth knowledges by highlighting differences. Compounding this impression, comparing associated with the theme of logistics, the topical epitome of care-full tinkering in its attention to seemingly trivial, practical details: Kerry detailed different approaches to routine heartbeat monitoring, Mel and Sue compared their signatures on consent forms (below), and PBM participants discussed who, when, where and how to perform vaginal seeding after a planned Caesarean. Against a backdrop of normative, broad or abstract narratives about labour, comparing—especially alongside logistics—engaged with the physical reality of childbirth.

In classes, comparing often linked to early labour. All observed midwife-teachers elaborated how people would know when to go to hospital or call their midwife. They gave examples of various bodily events, such as diarrhoea and vomiting, frequency and quality of early contractions, different feelings based on baby's position, passing the cervical mucous plug, the timing and appearance of amniotic fluid, and more. This section of the class underscored the normal range and boundaries of early labour, summarised here by midwife-teacher Maria:

¨Maria: Did you notice that I said, you might, you might get that, you might you might you might you might you might. <Pause> All of you will start off labour differently. There is no set pattern. It'd be dead simple if there was. But there isn't. You'll all start off differently. Some of you! Won't go into labour at all! But that's for week three. This week, we are gonna go into labour. Yeah? You'll all start differently. You might get a show, you might not. Your waters might break, they might not! <Pause> The waters can break before labour, in labour, halfway through, at the end, or not at all! A baby can come out, in the bag of water. You might get a bit of D and V, and it's nothing to do with labour starting, it might be something you've eaten! <Laughs> So you'll all start off differently.¨

These comparisons performed several types of care. Firstly, conveying range in normal experiences of early labour could help people stay home and avoid repeated or too-early trips to hospital, which may impede labour and negatively impact birthing people and families. Further, keeping labouring women out of hospital as long as possible cared for other hospital staff and institutional structures by reducing strain on resources. Emphasis on non-attendance constituted a care-full absence that encouraged nonengagement with maternity services. At the same time, Maria's contrasting examples of specific bodily events could help parents (to help midwives) to recognise potential dangers. Early labour may not seem like the most critical part of childbirth, but extensive details by midwife-teachers highlighted the implications of this stage for mothers, babies, midwives and institutions.

Other notable thematic associations occurred between comparing and medical professionals, suggesting care-full knowledges around midwives, doctors and hospitals in all antenatal settings. In the next excerpt, PBM women discussed interactions with midwives around the routine practice of “fundus measurement”, which monitors baby's growth by measuring the pregnancy bump at each appointment. This example incorporates comparing along with story, quantitative knowledge, compromise and other knowledges:

Rosa: Yeah, I felt that. Like I was measuring fine and then someone else did it and suddenly I was measuring small, and they wanted to book me in for a growth scan, And I just said, like, I just feel like all your measurements are complete nonsense. And they said, “Ah, okay we’ll get back to you”. And they called back and had the first midwife re-measure me and she was like, “Oh it's fine!” But if I hadn’t questioned that, I could have gone in for growth scans and then they could have found something else and then –

Sofia: And it depends how your baby is lying, its position, and it can change all the time. Or it depends on you –

Kylie: I've had the same because I'm short, they're like, “Oh your baby is small”…

This exchange describes maternity care practices in detail. The women discussed the fundus measurement practice itself, which relies on physical details like who does it, the mother's body and the baby's position. Interpersonal compromise emerged as the first midwife re-measured Rosa in response to her query, offering a care-full alternative to binary rejection/compliance of the growth scan. By attending to the significant emotional—affective—effect of the fundus measurements and growth scans, Rosa, Sofia and Kylie also implied its “dark sides”, such as unnecessary intervention, emotional distress or loss of trust in carers.

Midwifery care aside, the act of sharing this knowledge composed another form of care work, which offered nuance, emotional and practical impacts, and broader expectations. Normalising worked alongside comparing, as Rosa contrasted interactions and practices of different midwives, while Sofia and Kylie corroborated her experience. By depicting a shifting range of material factors regarding fundus measurement in an antenatal group, participants provided credible information about variability, plus affective reassurance and validation. A collective explanation of “measuring small”—that it depends on baby, mother, midwife, etc.—worked athwart standard quantitative practices of fundus measurement or growth scans, not fully complying or rejecting, but contextualising these interventions.

The previous excerpts demonstrate how other practices overlapped with the knowledge technique of comparing. Rosa's comment relied on first-hand storytelling, like most instances of comparing, although more distant stories frequently also used contrast to encompass range in experiences. Comparing not only grounded broad or abstract narratives, it also complicated and diversified expectations. Quantitative knowledges correlated with comparing to highlight variation, such as how long labour might last (Maria: “10 to 18 h, would be normal … it could be 24”) or how other knowledge practices complicated quantitative information (e.g., in observed discussions regarding due dates, centimetres of dilation). Rosa's story also foregrounds how “comparing” and “compromise” often acted together, as differences often characterised interpersonal negotiations or changes of plan—common facets of maternity care and experience (31, 41, 45). Compromise offered an oblique response to contradictory information, which disputed dominant notions of control or subjectivity, rejecting subject/object binarism and (re)constructing a more entangled, fluid decision-making agent.

Potential dark sides to the knowledge-and-care practice of comparing arose in its associations with the theme of choice, foregrounding not just variation but also individualism. The ideal of the autonomous subject can empower *and* discipline people [e.g., (48, 61, 64)], especially as contrasting choices in classes limited to topics of pain relief and placenta delivery. Further, these moments obscured choices that caregivers did not readily offer, such as declining monitoring or induction. When comparing amplified some differential choices, it could deflect or silence others, simultaneously protecting against perceived risks, encouraging compliance and disempowering birthing people.

Altogether, the knowledge technique of comparing acted especially care-fully in the observed antenatal sessions. By emphasising details and differences among births—using story, logistics, other actors, quantitative knowledges, etc.—participants adjusted abstract expectations and grounded information in lived realities. Comparing also creatively engaged formal knowledges and narratives, not rejecting or reiterating, but adding complexity and context. Similarly, compromise often arose alongside comparing, as relational fluidity disrupted subject/object binaries and negotiated control provided alternative paths. At other times, comparing reinforced normative individualism in association with choice, which could empower, protect, normalise and oppress. Comparing cared multiply for birthing people, babies, birth knowledges and—especially in classes—medical care providers, institutional structures and sociocultural norms. In its ability to tinker and work athwart, to expand and delimit, comparing exemplifies how knowledge practices perform powerful care work.

#### Normalising collective alternatives

Comparing often worked in conversation with the knowledge technique of normalising, where participants reinforced sameness or gave supporting examples. Normalising marked one of the most common practices in teacher-led settings, and also appeared significantly in groups. In classes, this technique tended to strengthen formal knowledges, with midwife-teacher phrases such as “all women do this”, “this is what happens” or the frequent use of second-person (“you”) imperatives in descriptions of labour. These examples demonstrate the darker, protective/suppressive aspects of this care-full knowledge, making some lives easier by silencing deviance. In more collective contexts, normalising often disrupted dominant discourses. Midwife-teachers strongly normalised intuition in the rare occasions they discussed that lay knowledge practice, as seen in universalising comments regarding pushing (Justine: “Your body will just take over”; Olivia: “When the baby's, there, and, and the body's ready to push, you’ll just do it, whatever the midwife says”). Groups also normalised intuition regarding pushing [Kath: “You can’t not (push) if that urge is there”] and in general (Ada: “You know your body more than anybody”). Normalising intuition built credibility around this alternative, collective knowledge practice all settings, by care-fully grounding and contesting formal knowledges.

Another interesting link occurred in the data between normalising and loss of control. Chaos rarely emerged in teacher-led settings, and its boundaried, normalised framing—often with intuition—stood out regarding pushing or transition. In one NHS Homebirth class, midwife-teacher Kerry addressed the all-important issue of when to engage maternity services in early labour:

¨Kerry: Basically ring us when you're in labour? Or, you know, give us a heads up as well, if, you know, if you think actually, second baby. Is it anybody's second baby? <A few hands raised> Yeah, they can come really quickly. <Laughs> Very quickly. So yeah, you know, once you start to regularly contract, give us a ring. Don't think I'll wait and wait. One of our midwives, second baby, eh, she was like, “No, I can't go in, no, I'm a midwife, I can't go in, I can't go in.” And then she had the baby in the car park! <Laughs> Because it can happen, quickly!¨

By preparing women for the likelihood of a fast second birth, Kerry revised mainstream assumptions about labour lasting a long time. She also normalised the chaos inherent in a fast labour; even a midwife might end up birthing her baby in the car park. Other participants also correlated a fast labour with feelings of chaos, including Joanna's accidental unassisted birth (“It went from nought to 60 and I was like, oh my … I am not coping”), and my second birth described at a Homebirth group (“She was out in like 45 min … it was just bonkers”). Apart from offering practically useful preparation, expectations about loss of control could be affectively reassuring, for example as Claire's story helped others stay calm in the face of copious vomiting and diarrhoea. Normalising chaos accommodated this otherwise-taboo carnality (28), adding real-life details and alternative ideas about birth or feeling in control.

Compromise emerged as another association with normalising, including subthemes of interpersonal negotiation, change of plan and uncertainty (i.e., negotiated knowledge). Most midwife-teachers discussed how women should expect changes in behaviour, sensations, preferences and circumstances during labour: Maria noted, “Women change, in labour” regarding what they want from partners; Olivia described “a change in your mood” as a key feature of transition; Justine talked about changing sensations during transition and pushing; Sheila acknowledged the impact of the hospital environment on labour, as a “shift change … changes everything again”. Similarly in groups, facilitator Kate noted that going to hospital “affects a lot of women more than they realise”, and Ada suggested that change in baby's heartbeat “probably happens all the time but we’re just not listening in constantly”. As with chaos, normalising negotiated control felt like an attempt to accept the contingency and uncertainty that characterises childbirth (65), complicating hegemonic ideals and providing affective reassurance.

Normalising cared multiply for birthing women, babies, midwives and more. Midwife-led normalising of formal top-down knowledges could encourage compliance with guidelines, silence deviant perspectives and experiences, and protect midwives and intuitions. However, participants also normalised intuition, chaos and compromise. Reinforcing these aspects of childbirth built space for collectivity, alternatives and taboos, while tinkering with and working athwart formal knowledges and expectations. Further, normalising engaged affective-effective care by emotionally and epistemically supporting unconventional birth experiences.

#### Laughter: mediating, lightening, bypassing and making space

The role of humour in all antenatal settings appears time and again in this project, although it does not significantly feature in previous literature on antenatal preparation. This section analyses how this pervasive and powerful knowledge technique operates as a care practice. Thematic associations with humour included transition, pushing, chaos and selfhood, pointing to humour's ability to navigate tricky knowledges, concerns, embarrassment, psychophysical intensity and other taboos. Humour affectively lightened certain topics, helping tellers and listeners to express and bypass the inadequacy of speech and feelings of embarrassment, discomfort or ambivalence. Such emotional buoyancy did not feel disingenuous, as humour helped to convey tellers' unspeakable and multiple physical and emotional sensations during climactic moments.

Even in less extreme instances, humour tended to work alongside self-experiences. In excerpts evoking pleasure, humour conveyed unexpected, uncomfortable or difficult-to-express subjectivities. Other examples include midwife-teacher stories about hallucinating on Entonox as “brilliant” or “enjoy[ing]” pethidine, and a group story about vocalising “*feeling so-o good*”. In one PBM anecdote about unexpected breastmilk let-down after a particularly good haircut, the room's laughter felt particularly poignant after the teller Monica's tearful story of her second baby in neonatal intensive care. The humour did not simply reinforce positivity, it also reflected and managed embarrassment at feeling self-love, and implicit taboos around carnality and pleasure—specifically in physiological links between sex, birth and breastfeeding. This excerpt and other instances emphasised the ability of humour to care-fully present embodied depictions of childbirth, encompass emotional dimensions and disrupt taboos and assumptions.

¨Humour performed a similarly multiple and care–full function regarding pain and negative emotions. Tellers and listeners constructed distressing interludes as humorous: E.g., Ella's dishevelled state due to uncontrolled vomiting, Claire's similar incident (“Everything's coming out of every orifice, at once <Laughter>”), Cherline's parody of her excruciating afterpains, distress at slow dilation from Sana and Mel, discussions about the potential trauma of vaginal examinations or epidural consent forms, and even anger at the patriarchy. Humour helped to mitigate negativity, allowing tellers and listeners to express the inexpressible and/or bypass the uncomfortable. A fairly typical class–based example follows, as attendees introduced themselves by stating one thing they worried about, and one thing they looked forward to about birth:

¨Susanna: What are we afraid of, mainly everything, a little bit. <Laughs> Em, how it starts, how long it takes, you know. (Maria: Exactly what we're gonna cover this week! So that's gonna be one worry dealt with Susanna) Yeah, well, probably gonna get more worries! <Laughter> Yeah, it is, we don't know what to expect. (Maria: Yes there's no, there's no handbook is there) Yeah, no, no.¨

Susanna's initial laughter bespoke self-deprecation and embarrassment about being “afraid” about “everything”. The fact that laughter spread when she expected “more worries” suggested that other attendees shared negative feelings about the uncertainty of childbirth *and* anxiety around those feelings. Many passages coded for uncertainty overlapped with humour, but only in classes, where uncertainty more directly contradicted normative, institutional knowledges (42). This excerpt reaffirms the role of humour in (effectively) addressing and (affectively) responding to taboo subjects. As elsewhere, laughter offered reassurance and protection, as well as deflection or partial silencing of concerns. In its links with self-experience, humour enables collective impulses to make light of subjectivity, navigate discomfort around psychophysical intensity, deflect and make space for taboo topics.

##### Humour and partners: working athwart gendered power dynamics and more

This analysis of humour as knowledge-and-care work gives special consideration to one of the most striking thematic intersections in the data: humour and partners. Across the transcripts, people used humour around half the time they spoke about romantic partners. Concerns abounded for many antenatal participants, and (usually male) partners were an easy target for jokes to lighten the mood. Previous research suggests other reasons. Mainly women ran and attended all the observed antenatal preparation sessions, with men as outsiders in the metaphorical and physical birth room (28). The fact that almost all participants expected and spoke positively about the presence of male partners during labour marks a sea change in the UK since the mid-twentieth century, when men—especially in working-class communities—almost never attended births (65). Despite UK-wide contemporary acceptance in the UK (25), male birth partners have expressed feelings of anxiety, fear, disappointment, isolation or uselessness (65), and some midwives note they can inhibit labour or reinforce gendered power dynamics (66). The sociocultural context is fraught: Male outsiders recently accepted into female spaces amid unexpressed ambivalence (31, 65), all within a patriarchal medical system and society (28, 29, 51). In this context, partner-oriented humour in the data care-fully both disputes and soothes gendered power relations.

Many jokes about partners decreased male power in the female birth space, bolstering and challenging gendered norms. Postnatal women often laughed at their male partner's confusion in group-based birth stories, for example as Rosa comically depicted her male partner using a high, panicky voice, and recalled how he apparently forget to catch the baby. Class participants laughed at Sandra's “poor man … pale as anything” during her Entonox hallucination, Justine's suggestion that partners might “get hit” during transition and Maria's comment that a labouring women might say “Stop touching me! It's really annoying”. The affective position of these examples seemed crucial, putting worried people at ease, especially in class settings, and attending to the emotional dimensions of labour. At the same time, laughing at partners care-fully broached awkward truths about birth in a patriarchal society, the usefulness of some men during labour and whether male partners wanted to be there.

¨A delightful and indicative example of how humour cared for knowledge, birthing women, male partners and gender dynamics took place at a PBM group, where Arun gave “his version” of his partner's birth story. Laughter frequently arose, as his externally-focused telling elicited humorous and expressive details from Sana. When Arun observed that gas and air “seemed to work”, Sana added depth and humour, exclaiming “thank god!” and miming herself desperately inhaling Entonox. He spoke in detail about her appearance and his part during pushing, and Sana's comical interjections confirmed his account as well as the inadequacy of this telling: When Arun referred to Sana's pain as “intensity,” Sana elaborated: “I thought, I'm gonna share, this, I'm gonna share this experience with Arun, so I bit him, into his thigh, twice <Laughter>.” His deadpan rejoinder that the pain he felt confirmed that her contractions “were strong, feelings” brought more laughter, as did Sana's nonapology (“In the back of my mind I was like, I should probably say sorry. <Laughter>”). Humour tinkered with Arun's description, communicating strong physical sensations, emotions and other verbally inexpressible details of Sana's birth. At the same time, her jokes conveyed scepticism around Arun's role amid her vocal appreciation of his support, and staunch affirmation of her epistemic authority on this topic.¨

Despite its absence from previous antenatal learning literature, humour emerged as one of the most care-full knowledge practices, creatively addressing complicated yet crucial aspects of birth like chaos, self-experience and partners. This latter topic engaged several facets of humour's knowledge-and-care work, as jokes clarified what men (should) practically do in the birth room, worked athwart expectations around their presence during labour, and exposed potential dark sides of male caregivers amid gendered power relations. Humour performed affective-effective work in many contexts, revealing emotional dimensions and non-normative physical sensations. By turning a fringe position, contradiction, worry or extreme experience into a joke, participants could approach a difficult topic without fully confronting it. This indirect tactic helped build new narratives, including multiplicity or ambivalence. The emotional effects of humour could reassure and uplift, and also—often simultaneously—increase compliance or deflect attention from concerns. Humour exhibits how collective practices care with complexity for birth knowledges, parents, partners, practitioners and power dynamics.

##### The careful ambivalence of silencing: resistance and protection (for whom?)

Intentional and enforced silences in birth knowledge demonstrate how absences care productively while potentially reinforcing norms and taboos. Excerpts coded for 'silencing' marked where participants bypassed concerns or disengaged from a session, sometimes linking to birthgiver control or resistance to authority. Additional silencing emerged during analysis, where topics, knowledges or patterns occurred in one setting but not another. I also identified absences by comparing information in sessions with stories from non-research settings, my own births and norms in literature (28, 29, 31). Scholars identify longstanding taboos around birth, such as silencing of carnality, sex, pleasure or pain (29, 31, 51). Western medicine also treats loss of control or “the unknown” as taboo (28, 29, 51, 67), and essentialised notions of motherhood can exclude feelings of negativity (18, 29, 44, 68). This investigation into silence relies on participant observation, personal experience and wider literature, although previous research does not specifically interrogate the complex role of intentional silencing in antenatal settings.

¨Some silences appeared in the rare coding of certain topics in certain settings. Absences in teacher-led classes reflected social and institutional norms (28, 41, 65), with a dearth of references to chaos, subjective resistance or emotionality. Groups rarely addressed risk or complex interventions, suggesting an impulse to bypass biomedical facets of birth. Care-full silences protected (some) people from (some) harm by reproducing setting-specific dominant discourses [e.g., (22, 61, 62)]. However, collective approaches more often broke normative taboos [e.g., (51, 65)], as themes of uncertainty, unknown and chaos emerged in group-led settings. Taboo negative emotions toward baby arose in groups, as Ada talked about difficulty bonding with her second baby (below), Sana described her first postnatal hours as “pure stress” due to difficulty feeding and lack of sleep, and Claire recalled thinking, “It's a good job you're, so gorgeous, because you'd be in the bin otherwise <Laughter>¨. Group discussions more often represented intense carnality, such as diarrhoea and vomiting, comparing a baby to “a three kilogram heavy poop!”, vaginal microbiomes or pushing sensations that included sensory pleasure. These examples creatively engaged taboos by emphasising material and affective realities, while classes more care-fully maintained silences, perhaps to avoid psychosocial discomfort and protect biomedical norms.¨

Regarding pain and negative emotion, transcripts included a dearth of references and explicit silencing. Some group participants avoided naming pain as such, as in Sana and Arun's story, reinforcing “natural” birth narratives that seek to denaturalise notions of childbirth pain (29, 68). However, this care-full silence accompanied storied and other in-depth descriptions of pain that made space for extreme and varied corporeality in groups. Class-based silences around pain appeared when participants implied without explicitly recognising pain or harm. Midwife-teachers tended to normalise multiple complex interventions by focusing on clinical procedure rather than decision-making opportunities or potential side effects; one diminutively described a Caesarean scar “like a smile”. Some participants dismissed explicit concerns, as in this NHS class example on how to administer the analgesia Remifentanil:

Cassie: That sounds horrendous. (Olivia: Yeah?) So you're on oxygen. You’re stuck to the bed, you're on a catheter.

Anna: Works very well though apparently? <Big laughter>

Olivia: It's very effective pain relief.

Midwife Olivia's interjection (“Yeah?”) immediately questioned Cassie's negative evaluation of Remifentanil as a highly invasive intervention. Laughter seemed to further deflect her concern, as other attendees re-focused on the drug's ability to relieve pain. Cassie did not appear reassured, but the class moved on to discuss another intervention, silencing Cassie's distress about side effects as unworthy compared to overriding concerns about pain. This care-full deflection could help some birthing people, invalidating one cause for concern (side effects) to offer a solution for another (pain). More clearly, silenced pain in classes cared for biomedicalised birth norms, encouraging compliance, strengthening taboos about pain, devaluing physiological labour and decentring birthing subjects.

The suppressive/protective aspect of silencing also existed in gaps in teacher-led classes, such as the sparse use of collective knowledges regarding more invasive medical interventions. Regarding nonmedical and simpler techniques, collective practices—stories, intuition, comparing, humour—afforded space to ground, add nuance, evoke emotions and disrupt standardised expectations. Midwife-teachers compared personal observations of TENS machines, told jokes about Entonox and linked intuition to active birth techniques (e.g., Justine above). However, more complex interventions (e.g., diamorphine, epidural, induction, instrumental or Caesarean births) rarely involved collective knowledges, relying on procedural knowledge with some explaining and demonstrating. Presenting complex medical interventions as unproblematic or non-negotiable could help birthing parents avoid the burden of decision-making (22, 61, 64), while compliant patienthood almost certainly protects midwives, doctors and institutions (10, 23). At the same time, by rendering certain topics as inaccessible to lay engagement, this epistemic silence could reinforce medicalisation and suppress alternative preferences or experiences.

Some silencing emphasised the protective capacity of care-full absences. When participants disengaged in teacher-led settings, they accessed one of the few knowledge practices available to them. The refusal of knowledge-as-care on biomedical terms [e.g., (61, 64)], in part, may be a form of resistance. Class attendees rarely responded verbally to descriptions of complex medical interventions, and sometimes admitted intentional ignorance:

¨Maria: Em, any worries? <Pause> Anything you're anxious about?

Isaiah: <Pause> Just.

Maisie: The labour I suppose. We try not to think about that too much though. <Laughs>

Maria: So that's what we're going to make you think about tonight. <Laughter>¨

This excerpt typifies many participants’ professed perspective on childbirth. Interacting with birth knowledges often meant thinking about unwanted outcomes; avoiding this information could be protective. Social maxims include “knowledge is power”, but also “ignorance is bliss” and “what we don’t know, can’t hurt us”. Wilful, care-full silences reflect a protective/suppressive impulse toward birth knowledges.

Groups displayed more intentional associations between silence and resistance. Jade muted her social media to avoid negative stories, Esther told other participants not to watch *One Born Every Minute*, and Nikita justified her decision to free-birth (birthing without professional medical assistance). Humour often featured in stories of silence and resistance, as in this PBM exchange:

¨Mel: I ended up having an epidural … after I'd say 50 h … And then I have to sign away, my life on this sheet … Yeah, “It can paralyse you, you might have seizures because if they drain too much fluid, spinal fluid off, you are going to crash”, all this stuff. And you're thinking, oh my god, it's got to this, all these things are going to now happen as well? Sue: I just didn't read it. <Laughter> (What!?) I couldn't handle, I can't say that I didn't sign it, my signature will be on it somewhere. It must be, because I had it! But.

Esther: But you didn't have capacity.

Sue: <Laughs> It wouldn't look like mine, it would just be like squiggles.¨

In the full transcript, the women spoke at length about difficulty managing consent processes in the middle of labour. This discussion revealed the affective impact and potential dark side—recognised in other research (64)—of a seemingly benign institutional detail (signing a form). Elaborating the ramifications of this logistical act was a moment of affective tinkering, where people offered concrete solutions to help others avoid Mel's distress. Sue's nonengagement marked a careful absence in her knowledge; her act of not-reading and—even more so—telling other women about it, disrupted and resisted idealised notions of choice and informed consent [e.g., (61, 64, 65)]. She challenged expected behaviour, and advocated something rarely voiced but often implied by participants regarding birth knowledge: Sometimes it felt better not to know, or impossible to understand. Using story, silencing and humour, this exchange care-fully embodied and disputed expectations around informed consent, including acknowledging the value of disengagement, and offering emotional buoyancy at an upsetting time.

Intentional and enforced silences appeared as important collective knowledge practices that downplayed, ignored and resisted potentially distressing aspects of birth. Most care-full absences also had dark sides that could reinforce dominant discourses or deny people beneficial knowledges. Ambivalent gaps in knowledge cared for some birthing people, and especially for medical professionals, institutions and sociocultural norms. Group-led settings and storytelling maintained some silences, but also worked athwart conventional taboos, grounding and obliquely addressing difficult topics like chaos, negative emotion or pain.

### First-hand storytelling: engaging multiplicity and performing care

Even without explicitly focusing on storytelling—the primary collective practice observed in antenatal sessions—stories have appeared throughout this analysis. The final section explores the most compelling story heard during observations, and by some definitions perhaps one of the least “careful”. Facilitator Kath later referred to it as “pretty, harrowing”, saying, “If it had been a different group I probably would have <mimes cutting motion> shut that down”. But Ada's story was full of care—tinkering, working athwart, effecting and affecting, creating absences and attending to darkness—for herself, pregnant and postnatal people, midwives, birth, birth knowledges and more. Like many in-depth first-hand stories, this tale employed the other collective knowledges considered here, including intuition, formal knowledges, comparing, normalising, humour and silencing. Ada's story offered a rich summary of how women used collective knowledges in antenatal sessions to care-fully construct birth knowledges.

The story emerged as part of an extended round of introductions, in which each woman described her previous birth(s) and reasons for attending the PBM group. Ada described how her four births were all “exactly, the same”, and all outside expected norms as once she reached five centimetres, “it took me 20 min to have a baby”. This unconventional claim surprised other attendees, demonstrating how stories often normalised difference—i.e., using comparing alongside normalising—to adjust assumptions about the normal progress of labour. At this point Ada elaborated the details of her traumatic second birth, relying primarily on story, comparing and a bit of humour. She began by contextualising her own behaviour as “dead chilled …. I love giving birth”, in laughable contrast to the midwife who was shouting “No, no, no … going a bit mad”. As elsewhere in the data, comparing worked to add materiality, emotionality and nuance, emphasising range in lived psychophysical experiences.

Sadly, the story turned from a light-hearted account of an obstinate “old … matron type” midwife to a dark tale of obstetric violence. Listeners gasped as Ada recalled the midwife's command (“Give her pethidine!”) and opiate injection without knowledge or consent, and Ada questioned whether she should continue her telling (“Do you want to know horrible –?”). Kath encouraged the story, rejecting and sidestepping its depiction as “horrible” by asking Ada to focus on “what is actually helpful”. When Ada replied “it does come out a nice story in the end”, she seemed to decide to recount her entire birthing history, with the intention to demonstrate the (affective-effective power) of intuition (“It's good to be aware of, you know your body more than anybody”). Unfortunately, no one listened to Ada or her intuition during her second birth, and she conveyed extreme distress at the disconnect between her intuition (“I can’t help but pushing”) and her caregiver's commands (“I couldn’t push”). Birthing her baby in a traumatised, unknowingly drugged state, Ada hallucinated the death of her child, serious organ damage, the death of the woman in the next bay, and a conspiracy to give her that woman's child. Procedural understandings of labour overruled the knowledge-and-care practice of intuition to devastating effect.

The impacts of this segment of the story relied heavily on context, including the safety and supportiveness of the PBM group, Ada's longer narrative and broader sociocultural norms. Non-consent and ignorance are familiar for too many birthing people, and some scholars characterise these epistemic injustices as obstetric violence (69). Ada's distressing account also included an affective pressure valve at one point, as other attendees laughed at her joking summary of her hallucinations: “I figured all this out”. Otherwise humour was nowhere to be seen in this section of the story, although it arose strongly later in the tale. Storytelling enacted the main knowledge-and-care effort, as Ada conveyed the holistic physical, emotional and epistemic trauma of obstetric violence through her detailed, situated recounting.

Ada performed some affective repair as she continued. She explained one source of her trauma as the mismatch between intuition and professional instruction. Her personal recovery also relied on speaking with a trusted midwife, reinforcing relationality of care and contrast in caregivers, and input from her clinical notes and awareness of side effects of pethidine facilitated understanding. Some silence and uncertainty remained over whether she had healed from this trauma, as her professed “little bit of, post-natal depression” felt like an understatement. Ada incorporated multiple knowledge practices to care for herself and listeners, including affective support from caregivers, uncertainty about the effects of her trauma, and intuition as an alternative to biomedical maternity care.

However, Ada's primary repair—and care—work appeared in her telling of subsequent births, whose joyful recounting formed a counterpoint to her previous experience. The brief account of her third birth emphasised how she created gaps in her care to avoid conflicting messages or other unwanted input. She kept midwives out of the room (“Go out! I’m fine”) although she did not fully refuse to engage (“I’ll shout ya if I need ya”). This absence made space for her to attend to her intuition, which brought real affective delight (“It were beautiful”). Ada's fourth birth utilised humour, contrast and silences to further heal and disrupt the negativity of her tale. She evoked shocked laughter at her first reference to sexual pleasure (“the fourth birth, I was like, we can take this a step further. And we did the orgasm thing”), especially with proximity to birth and her father's presence on the ward (“…and my dad was there. <Big laughter> Not for the orgasm bit!”). More humour arose as she imitated her father and partner using a deep voice and broad regional accent, and dramatic contrasts between her labour and their focus on sports news inspired renewed laughter:

¨Ada: I'm like, ohh, let's just go home, it's wind, it's got to be wind! <Laughter> It's not happening, is it? It's just, and then I go, <inhales> it's coming again. <Exhales, whispers> Oh, I don't know. It's not, it's, wind. <Deep voice> Come on, we'll go home. And then we went to t' television room, they were like, “Um, well, Match o't' Day's starting now. We'll, watch Match o't' Day and then we'll go home.” So I'm sat there thinking, I'm not watching Match o't' Day, I'm gonna go in this room on my own, and do this orgasm thing. <Laughter>¨

Uncertainty emerged around intuition, but Ada ultimately found space for her intuition, “did the orgasm thing” and, with some surprise, quickly birthed her baby. Silences, humour, comparing and intuition all featured in Ada's story-based resolution, performing complex care for teller and listeners.

Ada's first-hand, face-to-face story, told in a supportive context, depicts how even an apparent horror story of obstetric violence provided care-full birth knowledge. Empirically grounded details adjusted assumptions and depicted contrasting, fluid engagements with formal knowledges, intuition and care providers during childbirth. By turning a “horrible” story into “something helpful”, Ada worked athwart binarist birth discourses [e.g., (28, 29, 65)] and offered a holistic, integrated, diverse portrayal. Storied drama and humour affectively mediated the pain and pleasure in her story, and reflected crucial emotional resonances of the knowledge practices and physical sensations she described. Absences in her narrative held care-full spaces for uncertainty and unfinished healing from trauma, while the gaps she built in her maternity care provision offered her space for self-care and engagement with intuition. (If her previous midwife had been able to maintain such a care-full gap in her attentions, Ada may have avoided much trauma!) Another telling of this story might reinforce disempowerment, conflict, violence and enact a darker sort of care, but embedded in a group-led antenatal preparation session, Ada's account cared deeply and multiply for herself, those of us privileged to hear her tale, and—if we are care-full—those to whom we might pass her story.

## Conclusion

Public crises in maternity care call for better antenatal preparation (1, 24, 25) and for institutions to listen to birthing people (1–4, 8). *Knowing Childbirth* proposes care-full, collective learning as potential solution, elaborating on long-standing recommendations for participant-led antenatal preparation (12). Storytelling, group-led discussion and other collective birth knowledge practices address some of the shortcomings of conventional antenatal classes identified in previous research (12, 15, 16, 18, 21, 22, 24–26), building peer relationships while sharing knowledge that is more culturally appropriate, accessible, wide-ranging, grounded in lived experiences and centred on the birthgiver.

Framed by feminist critical literature on care (47–49), this paper demonstrates how unusual collective practices such as storytelling in classes, “distant” stories and intuition attended to gaps in conventional antenatal preparation. Techniques for navigating knowledges—namely, comparing, normalising, humour and silencing—also cared flexibly in groups and classes. Finally, one in-depth first-hand story depicts how the powerful and commonplace collective practice of storytelling utilises other knowledge practices and enacts complex knowledge-and-care work.

Recalling notions of radical mothering (44), collective birth knowledges perform grounded, creative and revolutionary care that resists disciplining conventional discourses. Stories, intuition and contrast emphasise nuance in lived experiences and tinker with abstract expectations. Sociomaterial contexts adjust the epistemic meanings of birth stories and other collective practices, and affective-effective resonances depend on tellers, audience or setting. Humour and stories evoke emotional dimensions to birth and knowledge, including space for psychosocial differences and ambivalences. All practices explored here work athwart dominant narratives that discipline birthing people, adding empirical information, re-valuing intuition, contextualising formal knowledges, using humour to disrupt assumptions or disengaging. Care-full gaps appeared in telling stories at a distance, exposing uncertainties, silencing concerns or resisting biomedicalisation. These absences could also indicate a dark side to knowledge-as-care, bolstering taboos, silencing undesirable outcomes or encouraging intuitional compliance. Through embodiment, emotional resonance, indirect involvement, meaningful absences and ambivalent multiplicities, collective antenatal learning has the power to care for birthing people, midwives, families and more.

## Data Availability

The datasets presented in this article are not readily available, however anonymised transcripts may be provided to other researchers from universities, NHS organisations or companies involved in health and care research in the UK or abroad. Research must concern maternity services or knowledge production, and be in accordance with the UK Policy Framework for Health and Social Care Research. Requests to access the datasets should be directed to leah.dequattro@manchester.ac.uk.
