# Correction: Antenatal preparation as care: birth stories and collective learning at work

**DOI:** 10.3389/fgwh.2025.1708942

**Published:** 2025-12-12

**Authors:** Leah De Quattro

**Affiliations:** Centre for the History of Science, Technology and Medicine (CHSTM), The University of Manchester, Manchester, United Kingdom

**Keywords:** birth stories, antenatal preparation, childbirth education, care, collective knowledge, feminist technoscience, socio-narratology, group-led

In the published article, the non-verbal data was missing from participant quotes and transcripts—namely “Laughs” “Laughter” and “Pauses”. These have been added as the article's analysis specifically addresses humour and silence in the data.

The non-verbal marker “<Laughter>” was excluded from a participant transcript.

A correction has been made to the section **Results and discussion**, *Uncommon collective practices: complementing standard knowledges*, *Class stories: materiality, affect and disrupting expectations*, paragraph 2:

¨Justine: When you suddenly get contractions, and pressure in your back passage, you don't like it. Okay? And for about 5 min, a lot of women, lose it. Just because, they, they are not used to the sensation of both together … And if you're a birth partner within punching distance you'll get hit. <Laughter>”  … It's very scary for the woman, but after a couple of minutes, it's over … And then you suddenly go, <clicks fingers> push! … You're so focused on pushing, it takes over your whole body and you forget about all the pain.

The non-verbal marker “<Laughs>” was excluded from a participant transcript.

A correction has been made to the section **Results and discussion**, *Uncommon collective practices: complementing standard knowledges*, *Class stories: materiality, affect and disrupting expectations*, paragraph 6:

¨Olivia: All a bit fuzzy? Ah, maybe. I'd say more so with pethidine than with diamorphine and that's the reason it changed, actually, so. Eh, generally we find that we prefer diamorphine, pethidine made everybody a bit woozy. But I had it with my, my first is a bit older and em, I enjoyed pethidine, I have to say <Laughs> I really like it! But, you know, everybody's different.¨

The non-verbal markers “<Laughs>” and “<Assent>” were excluded from a participant transcript.

A correction has been made to the section **Results and discussion**, sub-section *Intuiting labour: embodied, oblique knowledges*, paragraph 7:

¨Kath: [My friend] said to me, one thing I'll say to you now, “Don't let them tell you not to push.” She said, “I’m sure the reason I had a good back-to-back birth was because I didn't have some dickhead midwife telling me not to push.” <Laughs> <Assent> And as soon as my midwife walked in for my birth, and it was a midwife I'd met prior, I went, don't tell me not to push! Because I'm going to push! And I’ve been told I'm not allowed to – you know, straight off, I'm gonna push, I'm gonna push. Because you can't not if that urge is there.¨

The non-verbal markers “<Pause>” and “<Laughs>” were excluded from a participant transcript.

A correction has been made to the section **Results and discussion**, *Navigating with care: comparing,normalising, humour and silencing*, *Comparing: tinkering, working athwart and individualising*, paragraph 3:

¨Maria: Did you notice that I said, you might, you might get that, you might you might you might you might you might. <Pause> All of you will start off labour differently. There is no set pattern. It'd be dead simple if there was. But there isn't. You'll all start off differently. Some of you! Won't go into labour at all! But that's for week three. This week, we are gonna go into labour. Yeah? You'll all start differently. You might get a show, you might not. Your waters might break, they might not! <Pause> The waters can break before labour, in labour, halfway through, at the end, or not at all! A baby can come out, in the bag of water. You might get a bit of D and V, and it's nothing to do with labour starting, it might be something you've eaten! <Laughs> So you'll all start off differently.¨

The non-verbal marker “<Laughs>” was excluded from a participant transcript.

A correction has been made to the section **Results and discussion**, *Navigating with care: comparing,normalising, humour and silencing*, *Normalising collective alternatives*, paragraph 3:

¨Kerry: Basically ring us when you’re in labour? Or, you know, give us a heads up as well, if, you know, if you think actually, second baby. Is it anybody's second baby? <A few hands raised> Yeah, they can come really quickly. <Laughs> Very quickly. So yeah, you know, once you start to regularly contract, give us a ring. Don't think I'll wait and wait. One of our midwives, second baby, eh, she was like, “No, I can't go in, no, I’m a midwife, I can't go in, I can't go in.” And then she had the baby in the car park! <Laughs> Because it can happen, quickly!¨

**Text correction**

The non-verbal marker “<Laughter>” was excluded from a participant transcript.

A correction has been made to the section **Results and discussion**, *Laughter: mediating, lightening, bypassing and making space*, paragraph 3:

¨Humour performed a similarly multiple and care-full function regarding pain and negative emotions. Tellers and listeners constructed distressing interludes as humorous: E.g., Ella's dishevelled state due to uncontrolled vomiting, Claire's similar incident (“Everything's coming out of every orifice, at once <Laughter>”), Cherline's parody of her excruciating afterpains, distress at slow dilation from Sana and Mel, discussions about the potential trauma of vaginal examinations or epidural consent forms, and even anger at the patriarchy. Humour helped to mitigate negativity, allowing tellers and listeners to express the inexpressible and/or bypass the uncomfortable. A fairly typical class-based example follows, as attendees introduced themselves by stating one thing they worried about, and one thing they looked forward to about birth:¨

The non-verbal markers “<Laughs>” and “<Laughter>” were excluded from a participant transcript.

A correction has been made to the section **Results and discussion**,*Laughter: mediating, lightening, bypassing and making space*, paragraph 4:

¨Susanna: What are we afraid of, mainly everything, a little bit. <Laughs> Em, how it starts, how long it takes, you know. (Maria: Exactly what we're gonna cover this week! So that's gonna be one worry dealt with Susanna) Yeah, well, probably gonna get more worries! <Laughter> Yeah, it is, we don't know what to expect. (Maria: Yes there's no, there's no handbook is there) Yeah, no, no.¨

The non-verbal marker “<Laughter>” was excluded from a participant transcript.

A correction has been made to the section **Results and discussion**, *Humour and partners: working athwart gendered power dynamics and more*, paragraph 3:

¨A delightful and indicative example of how humour cared for knowledge, birthing women, male partners and gender dynamics took place at a PBM group, where Arun gave “his version” of his partner's birth story. Laughter frequently arose, as his externally-focused telling elicited humorous and expressive details from Sana. When Arun observed that gas and air “seemed to work”, Sana added depth and humour, exclaiming “thank god!” and miming herself desperately inhaling Entonox. He spoke in detail about her appearance and his part during pushing, and Sana's comical interjections confirmed his account as well as the inadequacy of this telling: When Arun referred to Sana's pain as “intensity,” Sana elaborated: “I thought, I’m gonna share, this, I’m gonna share this experience with Arun, so I bit him, into his thigh, twice <Laughter>.” His deadpan rejoinder that the pain he felt confirmed that her contractions “were strong, feelings” brought more laughter, as did Sana's non-apology (“In the back of my mind I was like, I should probably say sorry. <Laughter>”). Humour tinkered with Arun's description, communicating strong physical sensations, emotions and other verbally inexpressible details of Sana's birth. At the same time, her jokes conveyed scepticism around Arun's role amid her vocal appreciation of his support, and staunch affirmation of her epistemic authority on this topic.¨

The non-verbal marker “<Laughter>” was excluded from a participant transcript.

A correction has been made to the section **Results and discussion**, *Navigating with care: comparing, normalising, humour and silencing, Laughter: mediating, lightening, bypassing and making space, The careful ambivalence of silencing: resistance and protection (for whom?),* paragraph 2:

¨Some silences appeared in the rare coding of certain topics in certain settings. Absences in teacher-led classes reflected social and institutional norms (28, 41, 65), with a dearth of references to chaos, subjective resistance or emotionality. Groups rarely addressed risk or complex interventions, suggesting an impulse to bypass biomedical facets of birth. Care-full silences protected (some) people from (some) harm by reproducing setting-specific dominant discourses [e.g., (22, 61, 62)]. However, collective approaches more often broke normative taboos [e.g., (51, 65)], as themes of uncertainty, unknown and chaos emerged in group-led settings. Taboo negative emotions toward baby arose in groups, as Ada talked about difficulty bonding with her second baby (below), Sana described her first postnatal hours as “pure stress” due to difficulty feeding and lack of sleep, and Claire recalled thinking, “It's a good job you’re, so gorgeous, because you’d be in the bin otherwise <Laughter>¨. Group discussions more often represented intense carnality, such as diarrhoea and vomiting, comparing a baby to “a three kilogram heavy poop!”, vaginal microbiomes or pushing sensations that included sensory pleasure. These examples creatively engaged taboos by emphasising material and affective realities, while classes more care-fully maintained silences, perhaps to avoid psychosocial discomfort and protect biomedical norms.¨

The non-verbal markers “<Pause>”, “<Laughs>” and “<Laughter>” were excluded from a participant transcript.

A correction has been made to the section **Results and discussion,** *Navigating with care: comparing, normalising, humour and silencing, Laughter: mediating, lightening, bypassing and making space, The careful ambivalence of silencing: resistance and protection (for whom?)*, paragraph 8:

¨Maria: Em, any worries? <Pause> Anything you're anxious about?

Isaiah: <Pause> Just.

Maisie: The labour I suppose. We try not to think about that too much though. <Laughs>

Maria: So that's what we're going to make you think about tonight. <Laughter>¨

The non-verbal markers “<Laughter>” and “<Laughs>” were excluded from a participant transcript.

A correction has been made to the section **Results and discussion,** *Navigating with care: comparing, normalising, humour and silencing, Laughter: mediating, lightening, bypassing and making space, The careful ambivalence of silencing: resistance and protection (for whom?)*, paragraph 11:

¨Mel: I ended up having an epidural … after I'd say 50 h … And then I have to sign away, my life on this sheet … Yeah, “It can paralyse you, you might have seizures because if they drain too much fluid, spinal fluid off, you are going to crash”, all this stuff. And you're thinking, oh my god, it's got to this, all these things are going to now happen as well?

Sue: I just didn't read it. <Laughter> (What!?) I couldn't handle, I can't say that I didn't sign it, my signature will be on it somewhere. It must be, because I had it! But.

Esther: But you didn't have capacity.

Sue: <Laughs> It wouldn't look like mine, it would just be like squiggles.¨

The non-verbal markers “<Laughter>” and “<inhales>” were excluded from a participant transcript

A correction has been made to the section **Results and discussion**, *First-hand storytelling: engaging multiplicity and performing care*, paragraph 7:

¨Ada: I'm like, ohh, let's just go home, it's wind, it's got to be wind! <Laughter> It's not happening, is it? It's just, and then I go, <inhales> it's coming again. <Exhales, whispers> Oh, I don't know. It's not, it's, wind. <Deep voice> Come on, we'll go home. And then we went to t' television room, they were like, “Um, well, Match o't’ Day's starting now. We’ll, watch Match o't' Day and then we'll go home.” So I'm sat there thinking, I'm not watching Match o't' Day, I'm gonna go in this room on my own, and do this orgasm thing. <Laughter>¨

The original version of this article has been updated.

